# Effectiveness of Emerging Technologies in Physiotherapy for Women with Breast Cancer: A Systematic Review

**DOI:** 10.3390/medicina62040762

**Published:** 2026-04-15

**Authors:** Kyriaki Hadjiyiasemi, Christina Michailidou, Manos Stefanakis, Eleni Tolma

**Affiliations:** 1 Department of Health Sciences, University of Nicosia, Nicosia 2417, Cyprus; michailidou.c@unic.ac.cy (C.M.); stefanakis.m@unic.ac.cy (M.S.); 2 PASYKAF (Cyprus Association of Cancer Patients and Friends), Nicosia 2000, Cyprus; 3 Department of Primary Care and Population Health, University of Nicosia Medical School, Nicosia 2417, Cyprus; tolma.e@unic.ac.cy

**Keywords:** breast cancer rehabilitation, physiotherapy, virtual reality, telerehabilitation, exercise oncology, digital health

## Abstract

*Background and Objectives*: Breast cancer remains the most frequently diagnosed malignancy among women worldwide. As survival rates continue to improve, rehabilitation interventions focusing on functional recovery and quality of life have become increasingly important. Emerging technologies such as virtual reality, augmented reality, and telerehabilitation have recently been integrated into physiotherapy programs. This systematic review aimed to evaluate the effectiveness of technology-assisted physiotherapy interventions on quality of life, psychological outcomes, and functional recovery in women with breast cancer. *Materials and Methods*: A systematic search was conducted in PubMed, CINAHL Complete, and MEDLINE Complete for randomized controlled trials published between January 2010 and March 2026. Studies were included if they investigated exercise-based physiotherapy interventions incorporating technological modalities such as virtual reality, augmented reality, mixed reality, robotics, or telerehabilitation. Outcomes included quality of life, fatigue, pain, upper limb function, and psychological health. *Results*: Six randomized controlled trials involving approximately 398 participants (mean age range: 30–60 years) were included in the qualitative synthesis. The studies included women across different stages of the disease trajectory, including postoperative patients and long-term survivors. Interventions comprised virtual reality-based exercise programs, Kinect-based mixed reality systems, augmented reality telerehabilitation platforms, and internet-based rehabilitation programs. Across studies, significant improvements were consistently observed within groups in outcomes such as quality of life, upper limb function, pain reduction, and shoulder range of motion (e.g., *p* < 0.001). However, between-group differences were not consistently statistically significant, with several studies reporting comparable improvements in both intervention and control groups. *Conclusions*: Technology-assisted physiotherapy interventions may support functional recovery and improve quality of life among women with breast cancer. However, the available evidence remains limited, with important methodological constraints, as improvements were predominantly observed within groups, while consistent between-group differences were not demonstrated. Therefore, the comparative effectiveness of these interventions over standard rehabilitation remains inconclusive, highlighting the need for further high-quality randomized controlled trials.

## 1. Introduction

Breast cancer remains the most commonly diagnosed malignancy among women worldwide, according to the World Health Organization and the International Agency for Research on Cancer [1]. As survival rates improve, the focus of care has expanded beyond disease treatment toward long-term survivorship outcomes, including functional independence, emotional resilience, and overall quality of life [2]. Physiotherapy is a fundamental component of rehabilitation, aiming not only to restore physical function but also to improve quality of life and psychological well-being in women living with and beyond breast cancer [3,4].

Exercise-based physiotherapy interventions such as aerobic training, resistance exercise, flexibility training, and combined programs are widely recommended throughout the cancer continuum. Substantial evidence supports their safety and effectiveness in preventing and managing breast cancer-related lymphedema, reducing cancer-related fatigue, improving upper limb strength and mobility, enhancing functional capacity, and alleviating symptoms of anxiety and depression [5,6,7]. Beyond physical restoration, exercise contributes significantly to psychological health, improving self-efficacy, body image, and overall quality of life among breast cancer survivors [5,8].

In parallel with advances in exercise oncology, emerging technologies have introduced innovative modalities for delivering physiotherapeutic interventions. Technologies such as virtual reality (VR), augmented reality (AR), mixed reality (MR), immersive and non-immersive exergaming systems, robotic-assisted devices, wearable sensors, and telerehabilitation platforms are increasingly integrated into supportive cancer care [9,10,11]. These technologies aim to enhance patient engagement, provide real-time feedback, individualize exercise dosing, and facilitate remote supervision, thereby strengthening adherence to and continuity of rehabilitation programs [12,13].

Recent systematic reviews have begun to explore the clinical value of these approaches. For example, Tian et al. (2025) reported that technology-assisted interventions, particularly VR and exergaming programs, may improve fatigue, upper limb function, and quality of life among patients with breast cancer; however, these interventions were not consistently integrated with structured exercise protocols [13]. Similarly, Eid et al. highlighted that VR-based exercise can be an effective and safe adjunct to conventional rehabilitation, improving mobility, adherence, and well-being; however, their systematic review included heterogeneous oncology populations, such as breast, prostate, brain, and mixed cancer types [14].

Although these findings are promising, the existing literature is characterized by considerable methodological heterogeneity, variability in intervention protocols, and limited synthesis specifically examining the integration of technology-enhanced exercise interventions within structured physiotherapy programs for women with breast cancer.

Despite growing interest in digital rehabilitation approaches, an important research gap remains. Many existing reviews evaluate technology-based interventions as standalone approaches or include heterogeneous cancer populations without focusing specifically on exercise-centered physiotherapy in breast cancer. Moreover, the potential additive or synergistic effects of combining evidence-based exercise principles with immersive, robotic, or telehealth technologies on quality-of-life outcomes have not been comprehensively synthesized.

Therefore, the aim of the present systematic review is to evaluate the effectiveness of technology-enhanced exercise interventions on quality of life and psychological health in women with breast cancer across different stages of the disease trajectory. By systematically examining current evidence, this review seeks to clarify whether integrating technology-enhanced exercise interventions into structured physiotherapy programs provides additional benefits beyond conventional physiotherapy alone and to identify directions for future research and clinical application within oncology rehabilitation.

## 2. Materials and Methods

### 2.1. Eligibility Criteria

Studies were considered eligible if they were randomized controlled trials published between January 2010 and March 2026. The lower time limit (2010) was predefined a priori and justified as this period marks the transition of virtual reality (VR), robotics, extended reality, and other digital technologies from experimental or prototype systems to clinically applicable tools within structured exercise-based rehabilitation programs for women with breast cancer [10,15]. From 2010 onward, technological interventions increasingly incorporated standardized protocols, validated outcome measures, and clinically relevant endpoints, including physical function, upper limb performance, fatigue, quality of life, and psychological health [13,14,15,16,17].

Earlier studies (pre-2010) were predominantly feasibility or proof-of-concept trials, often characterized by small sample sizes, limited methodological rigor, heterogeneous intervention parameters, and inconsistent reporting of clinically meaningful outcomes [15,17]. Restricting inclusion to studies published from 2010 onwards therefore enhances methodological consistency, clinical applicability, and comparability across interventions while reflecting contemporary rehabilitation practice.

Additional inclusion criteria were: (1) peer-reviewed studies adult women diagnosed with stage I–III breast cancer; (2) structured exercise-based physiotherapy or rehabilitation intervention; (3) integration of a technological modality (e.g., immersive or non-immersive VR, augmented reality, mixed reality, robotics, digital rehabilitation platforms, tele-rehabilitation); (4) presence of an active control group; and (5) reporting of at least one predefined outcome of interest (quality of life, mental health outcomes, upper limb function, fatigue, or pain (Table 1)).

**Exclusion criteria**

- Studies including exclusively metastatic (stage IV) populations;
- Non-peer-reviewed publications (e.g., conference abstracts);
- Review articles and systematic reviews;
- Study protocols;
- Pilot or feasibility studies;
- Non-human studies;
- Non-English language publications.

### 2.2. Information Sources

A comprehensive literature search was conducted in three electronic databases: PubMed, CINAHL Complete, and MEDLINE Complete. The search covered publications from January 2010 through March 2026. These databases were selected due to their comprehensive coverage of biomedical, rehabilitation, physiotherapy, and nursing research. Reference lists of eligible studies were also screened manually to identify additional relevant trial.

### 2.3. The Search Strategy

The search strategy was developed independently and was not based on any previously published systematic review.

To ensure methodological independence, comprehensiveness, and up-to-date coverage, a de novo systematic search was conducted across PubMed, CINAHL Complete, and MEDLINE Complete from January 2010 to March 2026. These databases were selected due to their strong and complementary coverage of biomedical, clinical, physiotherapy, and rehabilitation research, which are highly relevant to the topic of this review.

Particular emphasis was placed on identifying primary studies published from 2023 onwards, in order to capture recent technological advancements that may not have been included in prior reviews. This approach minimizes potential selection bias associated with reliance on secondary sources and ensures that the present review reflects the most current evidence on technology-enhanced exercise interventions in breast cancer rehabilitation.

Although broader databases such as Scopus may provide wider coverage, they were not included as it was considered that the selected databases would be sufficient to identify the majority of relevant studies. This may have limited the identification of additional studies, and this has been acknowledged as a limitation of the search strategy.

The following search strategy was used in each of the three databases:

(“breast cancer” OR “breast neoplasm*” OR “mammary carcinoma”)AND(“rehabilitation” OR “physiotherapy” OR “physical therapy” OR “exercise”)AND(“virtual reality” OR “augmented reality” OR “mixed reality” OR “extended reality” OR “immersive virtual therapy” OR “non-immersive virtual therapy” OR “telerehabilitation” OR “digital rehabilitation” OR “technology-assisted” OR “robotics”)AND(“quality of life” OR “mental health” OR “depression” OR “anxiety” OR “upper limb function” OR “arm function” OR “fatigue” OR “pain”)AND(“randomized controlled trial”)

Filters applied were publication date (2010–2025) and study design (randomized controlled trials and clinical trials).

The full electronic search strategies for all databases are provided in the Supplementary Materials (File S1).

This systematic review was registered in the PROSPERO database (Registration number: CRD420261324338). Minor modifications to the protocol were made during the review process. The search strategy was reported in accordance with PRISMA 2020 guidelines.

Only randomized controlled trials (RCTs) were included in order to ensure a high level of evidence and methodological rigor. Pilot, feasibility, and non-randomized studies were excluded to maintain consistency in study design and comparability of outcomes.

### 2.4. Selection Process

The study was conducted in accordance with the Preferred Reporting Items for Systematic Reviews and Meta-Analyses (PRISMA 2020) guidelines [17]. All records identified through the database searches were exported to a reference manager (RefWorks), and duplicate studies were removed prior to screening.

Two reviewers independently screened the titles and abstracts of the identified studies to assess their eligibility based on the predefined inclusion and exclusion criteria. Studies that did not meet the eligibility criteria were excluded at this stage.

Full-text articles were retrieved for studies that appeared to meet the inclusion criteria or when eligibility could not be determined based on the title and abstract alone. The same two reviewers independently assessed the full-text articles for final inclusion.

Any disagreements between reviewers regarding study eligibility were resolved through discussion and consensus. When necessary, consultation with a third reviewer was used to reach a final decision.

**Figure 1:**
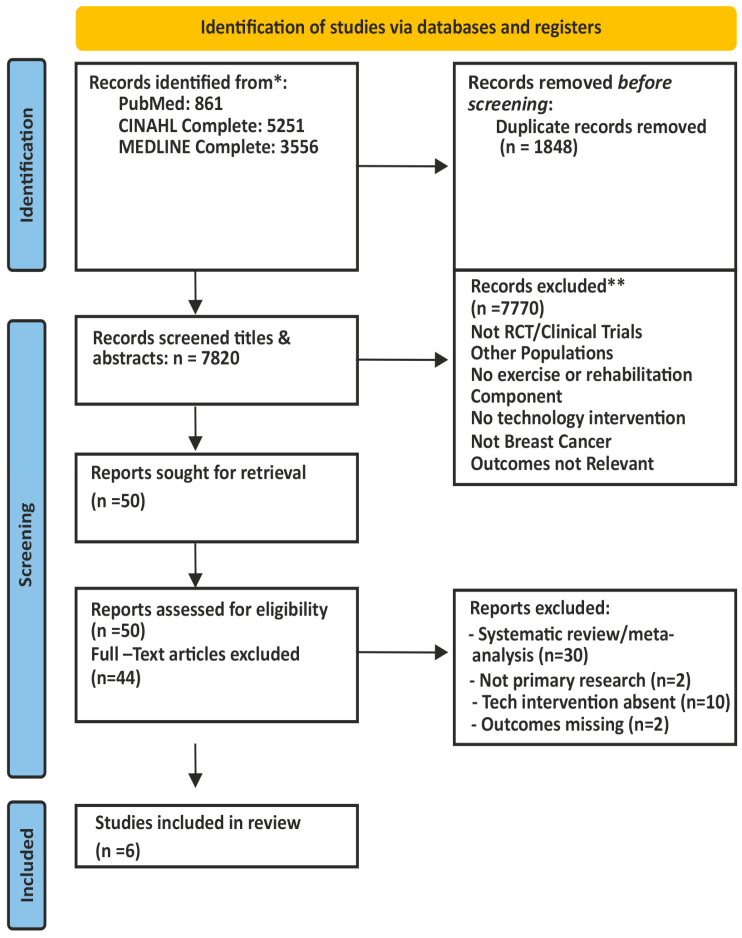
PRISMA flow diagram of the study selection process. * Search conducted in PubMed, CINAHL Complete and MEDLINE Complete databases. Search strategy included predefined keywords and filters. ** Records were excluded based on title and abstract screening according to predefined inclusion and exclusion criteria.

The overall study selection process is illustrated in the PRISMA flow diagram (Figure 1).

### 2.5. Data Collection Process

Data extraction was performed independently by two reviewers (KH & CM) using a standardized data extraction form (Table 2). Extracted data included study design, sample size, participant characteristics (age, cancer stage, treatment status), intervention characteristics (type of exercise, technological modality, duration, frequency, intensity), comparator characteristics, outcome measures, follow-up duration, and key findings. Any discrepancies were resolved by discussion to ensure data accuracy and completeness.

### 2.6. Data Items

Primary outcomes of interest were quality of life and mental health outcomes, including anxiety and depression. Secondary outcomes included upper limb function, arm mobility and strength, fatigue, and pain. Additional variables extracted included intervention duration, adherence rates, and type of technological platform used (e.g., immersive VR, non-immersive VR, robotics, tele-rehabilitation systems). Where reported, adverse events and safety outcomes were also recorded.

### 2.7. Risk of Bias Assessment

The methodological quality and risk of bias of the included studies were assessed independently by two reviewers using the Cochrane Risk of Bias 2 (RoB 2) tool, which is recommended for randomized controlled trials [18]. The RoB 2 tool evaluates potential sources of bias across five domains: (1) bias arising from the randomization process; (2) bias due to deviations from intended interventions; (3) bias due to missing outcome data; (4) bias in measurement of the outcome; and (5) bias in selection of the reported result.

Each domain was rated as “low risk of bias,” “some concerns,” or “high risk of bias,” and an overall risk of bias judgment was assigned to each study based on domain-level assessments [18,19]. The assessment was conducted independently by two reviewers, with disagreements resolved through discussion and consensus, and when necessary, consultation with a third reviewer.

The methodological approach of this systematic review was conducted in accordance with the Preferred Reporting Items for Systematic Reviews and Meta-Analyses (PRISMA) guidelines [17].

The results of the risk of bias assessment were summarized in both a tabular format and graphical visualization (Table 3).

Overall, most studies demonstrated a low risk of bias in domains related to the randomization process and missing outcome data. However, some concerns were identified in the domain related to deviations from intended interventions, primarily due to the inability to blind participants in exercise-based rehabilitation trials. In general, the overall methodological quality of the included studies was considered acceptable for inclusion in the qualitative synthesis.

### 2.8. Synthesis Methods

Due to heterogeneity in intervention protocols, technological modalities, duration of interventions, and outcome measures, a qualitative synthesis was conducted. Studies were grouped according to type of technology (e.g., immersive VR, non-immersive VR, robotics, tele-rehabilitation) and primary outcome domain. Where sufficient homogeneity existed, findings were comparatively analyzed to explore patterns of effectiveness across modalities.

**Table 2:** Characteristics of the included studies.

Study	Design	Sample (N)	Participants	Intervention	Comparator	Outcome Measures	Duration/Frequency/Intensity	Follow-Up	Key Findings
Basha et al., 2022 [6]	Single-blinded RCT	60	Women with unilateral BCRL, >30 yrs, ≥1 year post-diagnosis	Xbox Kinect VR exergaming (upper limb functional movements) + CDT (manual lymphatic drainage, compression, exercises); individualized game progression	Resistance training (dumbbells: rows, curls, bench press, triceps) at 50–60% 1 RM, 2 sets × 10–12 reps, progressive overload + CDT	VAS, DASH, ROM, muscle strength, SF-36 QoL	8 weeks, 5×/week	Post (8 weeks)	VR improved pain, DASH, ROM; resistance superior for strength
Feyzioğlu et al., 2020 [20]	RCT	40	Women post breast cancer surgery (30–60 yrs), receiving adjuvant therapy	Kinect-based VR exergaming (task-oriented upper limb movements) + massage + mobilization; 45 min/session	Standard physiotherapy (passive mobilization, massage, ROM exercises, home program)	VAS, ROM, grip strength, DASH, TKS	6 weeks, 2×/week	Post (6 weeks)	Significant within-group improvements; between-group differences significant for TKS (*p* = 0.001) and DASH (*p* = 0.025), but not for other outcomes.
Galiano-Castillo et al., 2016 [21]	RCT	81	Stage I–III breast cancer survivors post-treatment	Internet-based tailored exercise (aerobic + resistance + mobility), supervised remotely, individualized progression (ACSM guidelines)	Usual care (general physical activity recommendations)	QoL (EORTC QLQ-C30), pain, fatigue, strength	8 weeks, 3×/week (~90 min)	6 months	Improved QoL, fatigue, strength maintained
Lim et al., 2025 [22]	RCT	77	Stage I–III breast cancer survivors, ≥6 months post-treatment	Mixed reality exercise (KMR): HIIT-based aerobic + resistance bodyweight training, 40 s work/20 s rest, real-time feedback, progressive intensity	Home stretching: flexibility exercises (upper body & shoulder mobility), 40 s stretch/20 s rest	ROM, DASH, FACT-B, IPAQ	8 weeks, 3×/week, 30 min	Post (8 weeks)	Improved ROM, DASH, physical activity
Park et al., 2023 [23]	Multicenter RCT	100	Postoperative breast cancer patients (≤8 weeks post-surgery)	AR-based telerehabilitation (UINCARE): structured exercise (warm-up, main, cool-down), real-time feedback, progressive difficulty	Brochure-based home exercise (same exercises without feedback/monitoring)	ROM, QuickDASH, FACT-B, EQ-5D	8 weeks, daily	4, 8, 12 weeks	Both groups improved; no between-group differences
Ibrahim et al., 2024 [24]	Double-blind RCT	40	Stage I–II post-operative breast cancer women (20–60 yrs)	VR Pablo gaming + standard rehab: task-specific training + exercises (2 sets × 15 reps) + intermittent compression (60 mmHg, 15 min)	Standard rehab: ROM, strengthening, pendulum, pumping exercises (2 sets × 15 reps) + compression	Pain, fatigue, ROM, grip strength, ADL, anxiety	8 weeks, 3×/week	2 months	Significant improvements favoring VR group

**Table 3:** Summary of within- and between-group effects across included studies.

Study	Outcomes Assessed	Within-Group Effects	Between-Group Effects
Maged A. Basha., 2022 [6]	Pain, range of motion, strength, quality of life	Statistically significant improvements (*p* < 0.001)	Not clearly reported
Feyzioğlu et al., 2020 [20]	Pain, range of motion, strength, DASH, TKS	Statistically significant improvements (*p* = 0.001)	Mostly non-significant; significant differences observed only for selected outcomes (DASH, TKS)
Galiano-Castillo et al., 2016 [21]	Range of motion, quality of life, strength	Improvements observed	Not reported
Lim et al., 2025 [22]	Active and passive range of motion	Statistically significant improvements (*p* < 0.001)	No statistically significant differences (*p* > 0.3–0.5)
Park et al., 2023 [23]	Quality of life, fatigue, functioning	Statistically significant improvements (*p* < 0.001)	Not clearly reported
Ibrahim et al., 2024 [24]	Pain, function, range of motion, strength	Statistically significant improvements (*p* = 0.001)	Not clearly demonstrated

### 2.9. Reporting Bias Assessment

Potential reporting bias was assessed by examining discrepancies between study protocols (where available) and reported outcomes. Selective outcome reporting was considered when outcomes described in the methods were not presented in the results.

### 2.10. Certainty Assessment

The certainty of evidence for each outcome was evaluated using the Grading of Recommendations Assessment, Development and Evaluation (GRADE) framework. This approach assesses the overall confidence in the evidence by examining several domains, including risk of bias, inconsistency of results, indirectness of evidence, imprecision of estimates, and potential publication bias.

Because the included studies were randomized controlled trials, the certainty of evidence initially started at a high level. However, the certainty was downgraded due to methodological limitations, particularly risk of bias related to the lack of participant and personnel blinding, which is inherent in exercise-based rehabilitation interventions. In addition, substantial heterogeneity in intervention protocols and outcome measurement tools contributed to inconsistency across studies.

Imprecision in the estimated effect was also considered a reason for downgrading the level of evidence. Imprecision was assessed based on factors such as small sample sizes, a limited number of studies contributing to specific outcomes, and the resulting uncertainty around effect estimates.

After considering these factors, the overall certainty of evidence for most outcomes was rated as low, while the certainty for fatigue outcomes was rated as very low, primarily due to additional concerns related to imprecision and the limited number of studies reporting this outcome.

## 3. Results

### 3.1. Study Selection

The initial database search yielded 9668 records (PubMed: 861; CINAHL Complete: 5251; MEDLINE Complete: 3556). All identified records were exported and duplicate entries were removed prior to screening (Figure 1).

### 3.2. Study Characteristics

Several studies were assessed in full text but were excluded because they did not meet the predefined inclusion criteria. For example, the systematic review by Tian et al. (2025) investigated the effects of virtual reality interventions in women with breast cancer; however, it was excluded because it did not report primary randomized controlled trial data and focused primarily on symptom management rather than structured exercise-based rehabilitation interventions [13]. Similarly, Farrah et al. (2019) explored digital health technologies in cancer rehabilitation, but the study was excluded because the interventions did not include a structured exercise component [19].

Other studies were excluded due to differences in study design. For instance, Eid et al. (2025) was excluded because it was a systematic review rather than a primary randomized controlled trial [14]. Additionally, several studies were excluded because the population did not specifically include women with breast cancer or because relevant outcomes (e.g., quality of life, fatigue, upper limb function, or psychological health) were not reported.

In total, six randomized controlled trials met the inclusion criteria and were included in the qualitative synthesis [6,20,21,22,23,24]. These studies investigated the effects of technology-enhanced exercise or physiotherapy interventions in women with breast cancer or breast cancer-related lymphedema (Table 2).

The included studies demonstrated variability in terms of technological modality, including virtual reality (VR)-based interventions, Kinect-based mixed reality systems, augmented reality (AR) telerehabilitation platforms, internet-based exercise programs, and virtual gaming rehabilitation systems combined with conventional physiotherapy. Intervention durations ranged from 6 to 12 weeks, with some studies including follow-up assessments.

Sample sizes across the included trials ranged from 40 to 100 participants. Most interventions combined structured exercise programs with interactive or digital technologies designed to enhance engagement, provide real-time feedback, and support adherence. Control groups typically received standard physiotherapy, resistance exercise, home-based stretching programs, or usual care.

The primary outcomes evaluated across the studies included quality of life, pain intensity, upper limb function, shoulder range of motion, fatigue, psychological outcomes (e.g., anxiety), and functional independence. Measurement tools commonly used across the trials included validated instruments such as the EORTC QLQ-C30 [25], FACT-B, DASH or QuickDASH, as well as scales for pain (VAS or NRS), fatigue, and psychological health (Table 2).

The included studies were conducted in different clinical contexts, including postoperative rehabilitation, management of breast cancer-related lymphedema, and rehabilitation of breast cancer survivors following completion of adjuvant treatment. Most interventions combined structured exercise programs with interactive digital technologies designed to enhance patient engagement, adherence to rehabilitation protocols, and motivation through real-time feedback or gamified environments.

Across the studies, the primary outcomes assessed included quality of life, upper limb function, fatigue, pain intensity, shoulder range of motion, and muscle strength. Quality of life was commonly evaluated using validated patient-reported outcome measures, including the Functional Assessment of Cancer Therapy—Breast (FACT-B), the European Organization for Research and Treatment of Cancer Quality of Life Questionnaire (EORTC QLQ-C30), the Short Form Health Survey (SF-36), and the EuroQoL 5-Dimension 5-Level (EQ-5D-5L). These instruments assess multiple domains of physical, emotional, and social well-being that are particularly relevant for breast cancer survivors undergoing rehabilitation.

### 3.3. Synthesis of Results

Across the included studies, technology-assisted interventions were generally associated with significant improvements in clinical outcomes within groups, including pain, range of motion, muscle strength, physical function, and quality of life.

However, between-group differences were not consistently observed. In several studies, both intervention and control groups demonstrated significant improvements over time, without statistically significant differences between them. For instance, Lim et al. 2025 [22] reported significant within-group improvements in active and passive range of motion (*p* < 0.001), while no significant between-group differences were identified (*p* > 0.3–0.5).

Only a limited number of outcomes, primarily specific functional measures (e.g., DASH, TKS), showed statistically significant between-group differences. Overall, although the findings indicate that participants improved over time, the comparative effectiveness of technology-assisted interventions over standard care remains inconclusive (Table 3).

### 3.4. Reporting Bias

The potential risk of bias due to missing results arising from reporting biases was assessed qualitatively. Most studies appeared to report the primary and secondary outcomes described in their methodology sections, and no clear evidence of selective outcome reporting was identified.

**Figure 2:**
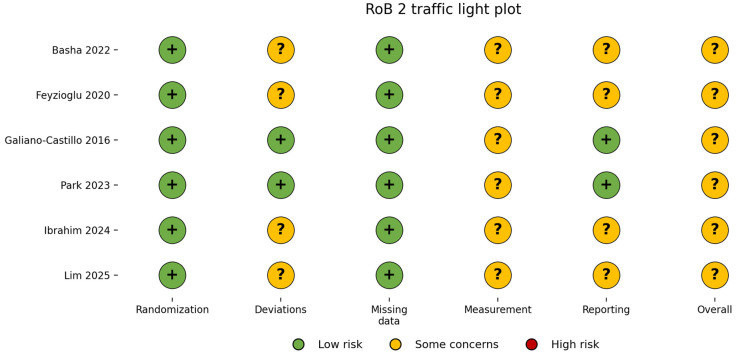
Risk of bias assessment using the RoB 2 tool [6,20,21,22,23,24].

The overall risk of bias across studies is presented in Figure 2 using the RoB 2 tool. Most domains were rated as low risk, particularly in relation to randomization and missing outcome data. However, several studies were judged as having some concerns, mainly in domains related to deviations from intended interventions and outcome measurement. No studies were classified as having a high risk of bias.

Overall, the included studies were considered to have an acceptable methodological quality, although the presence of some concerns across multiple domains should be taken into account when interpreting the findings.

### 3.5. Effects of Technology-Assisted Exercise Interventions

#### 3.5.1. Quality of Life

Four studies evaluated quality of life using validated instruments (FACT-B, EORTC QLQ-C30, SF-36, EQ-5D-5L) [6,21,22,23]. Improvements in quality of life were consistently observed in participants receiving technology-enhanced exercise interventions; however, these effects appear to be closely linked to specific characteristics of the technological modalities and exercise prescription.

In particular, interventions incorporating interactive virtual reality (VR) or mixed reality (MR) systems provided real-time visual and auditory feedback, enabling participants to monitor movement quality and performance. For example, VR-based exergaming platforms required continuous upper limb engagement through task-oriented activities (e.g., reaching, coordination-based tasks), which likely contributed to improvements in physical functioning domains of quality of life. Similarly, MR-based programs integrated high-intensity interval training (HIIT) structures (e.g., 40 s work/20 s rest) and adaptive progression based on performance data, promoting both cardiovascular and muscular engagement [22].

In contrast, interventions lacking feedback mechanisms (e.g., brochure-based home exercise) demonstrated less pronounced improvements [22,23], suggesting that feedback, engagement, and progression may be key mediators of quality-of-life outcomes, particularly in domains related to physical functioning and emotional well-being.

#### 3.5.2. Upper Limb Function

Five studies assessed upper limb function using DASH scores, grip strength, and functional performance measures [22,23]. Improvements in upper limb function were observed across most technology-enhanced interventions and were strongly associated with exercise specificity, intensity, and motor learning mechanisms.

VR-based interventions (e.g., Kinect systems) emphasized repetitive, task-specific upper limb movements (flexion, abduction, rotation), performed in an interactive environment that required coordination, timing, and accuracy [6,22]. This likely enhanced neuromotor control and motor relearning, contributing to reductions in disability scores.

Additionally, MR-based exercise programs incorporated combined aerobic and resistance training under structured HIIT protocols, with automatic adjustment of intensity based on user performance [22]. This individualized progression ensured that participants exercised near their functional capacity, potentially explaining improvements in both muscular strength and functional performance.

In contrast, conventional physiotherapy or unsupervised home programs lacked real-time feedback and objective monitoring, which may have limited optimal progression and motor engagement, resulting in smaller or comparable improvements [20,21].

Fatigue

Fatigue outcomes were evaluated in two studies [21,24], with both reporting reductions following technology-enhanced interventions. These improvements appear to be related to the integration of aerobic and resistance components and the regulation of exercise intensity.

Specifically, MR-based interventions applied interval-based training (high-intensity interval training, HIIT) and monitored repetition performance, enabling participants to maintain appropriate intensity levels while avoiding excessive fatigue [22,23]. Furthermore, remote or digital platforms allowed for structured scheduling (e.g., 3 sessions/week) and adherence monitoring, which may have contributed to consistent energy expenditure and gradual adaptation [23].

However, due to the limited number of studies and variability in intervention protocols (e.g., VR gaming vs. telerehabilitation), the mechanisms underlying fatigue reduction remain less clearly defined.

#### 3.5.3. Pain

Pain intensity was assessed in four of the six included studies primarily using the Visual Analog Scale (VAS) or the Numerical Rating Scale (NRS). Among these, three studies reported significant reductions in pain following technology-supported rehabilitation interventions whereas one study reported improvements over time in both groups without statistically significant between-group differences [6,20,21].

These effects may be partially explained by the interactive and engaging nature of technology-enhanced exercise. Virtual reality-based interventions may provide distraction through immersive environments, reducing pain perception via cognitive and attentional mechanisms. In parallel, structured exercise components—such as controlled repetitions, progressive overload, and task-specific training—may contribute to improved joint mobility and reduced musculoskeletal discomfort.

Additionally, interventions combining exercise with adjunct therapies (e.g., compression therapy or manual lymphatic drainage) demonstrated further improvements [6], suggesting that multimodal rehabilitation approaches may enhance pain reduction outcomes.

Within the included studies, both the Visual Analog Scale (VAS horizontal line ranging from 0 to 10) and the Numerical Rating Scale (NRS numeric scale from 0 to 10) were used to assess pain; however, none of the studies directly compared these tools. Therefore, no conclusions can be drawn regarding the superiority of one scale over the other based on the available evidence.

Nevertheless, both scales are widely accepted and validated in oncology populations. The NRS may be more practical in clinical settings due to its simplicity and ease of use, whereas the VAS may offer greater sensitivity in controlled research contexts [26].

Shoulder Range of Motion

Four studies assessed shoulder range of motion using goniometric measurements [24,25,26,27] reporting improvements in flexion, abduction, and overall mobility. These improvements were particularly associated with the type and execution of exercises facilitated by technological systems.

VR and AR-based interventions promoted active, repetitive upper limb movements across multiple planes, often embedded within functional tasks or game scenarios [20,23]. This encouraged a greater volume of movement compared to conventional therapy, potentially leading to enhanced joint mobility.

Moreover, some interventions incorporated progressive exercise difficulty and real-time corrective feedback, ensuring proper movement execution and gradual increase in range [22]. In MR-based systems, the inclusion of combined resistance and aerobic exercises further supported improvements in joint mobility by enhancing muscular strength and endurance.

In contrast, control interventions relying on static instructions (e.g., brochures) lacked these dynamic and adaptive features, which may explain the absence of significant between-group differences in some studies [23].

### 3.6. Heterogeneity of the Included Studies

Heterogeneity among the included randomized controlled trials was assessed to determine the comparability of the study findings. Variability was observed across the included studies in terms of technological modalities, intervention protocols, outcome measures, and participant characteristics [6,20,21,22,23,24].

The interventions differed substantially, including virtual reality-based rehabilitation, Kinect-based mixed reality exercise systems, augmented reality telerehabilitation platforms, and internet-based rehabilitation programs [6,20,22,24]. In addition, differences were identified in exercise protocols, intervention duration, and the timing of rehabilitation, as some studies focused on postoperative rehabilitation while others included breast cancer survivors who had completed their primary treatments.

Variability was also present in the outcome measures used to evaluate rehabilitation effectiveness. Quality of life was assessed using different validated instruments such as FACT-B, EORTC QLQ-C30, SF-36, and EQ-5D-5L, while functional outcomes were measured using tools such as the DASH questionnaire, shoulder range-of-motion assessments, grip strength measurements, and pain scales [21,22,23].

Due to this clinical and methodological heterogeneity, a quantitative meta-analysis was not considered appropriate. Therefore, a narrative synthesis approach was used to summarize the findings of the included studies.

In addition, it should be acknowledged that breast cancer represents a biologically heterogeneous disease. Variations in tumor characteristics, including emerging distinctions such as HER2-low and ultralow expression, may influence treatment response, recovery trajectories, and rehabilitation outcomes. This biological variability was not consistently reported across the included studies and may represent an additional source of heterogeneity affecting the interpretation of results [27].

### 3.7. Certainty of Evidence (GRADE)

The certainty of evidence for each outcome was evaluated using the Grading of Recommendations Assessment, Development and Evaluation (GRADE) framework (Table 4) [28]. The GRADE approach provides a structured method for assessing the overall confidence in the evidence by considering several domains, including risk of bias, inconsistency of results, indirectness of evidence, imprecision of effect estimates, and potential publication bias.

Because the included studies were randomized controlled trials, the certainty of evidence initially started at a high level. However, the certainty was downgraded due to methodological limitations and inconsistencies identified across studies. In particular, several studies presented concerns related to risk of bias, mainly due to the lack of participant and personnel blinding, which is inherent in exercise-based rehabilitation interventions and may influence subjective outcomes such as quality of life, fatigue, and pain [18].

In addition, substantial variability in intervention protocols, technological modalities (e.g., virtual reality systems, mixed-reality exercise platforms, and telerehabilitation programs), and outcome measurement tools contributed to inconsistency in the available evidence [6,20,23]. Differences in intervention duration and patient populations may also have influenced the magnitude of the reported effects.

The certainty of evidence was further downgraded due to imprecision, as several studies included relatively small sample sizes and a limited number of trials contributed to specific outcomes. This was particularly evident for fatigue, where only two studies were available [21,24].

After considering these factors, the certainty of evidence for outcomes related to quality of life, upper limb function, pain, and shoulder range of motion was rated as low certainty, while the certainty for fatigue outcomes was rated as very low certainty.

Overall, the GRADE assessment suggests that technology-enhanced physiotherapy interventions may provide beneficial effects for women with breast cancer; however, additional high-quality randomized controlled trials with larger sample sizes and standardized intervention protocols are needed to strengthen the current evidence base.

## 4. Discussion

The present systematic review examined the effectiveness of technology-enhanced exercise interventions in women with breast cancer, indicating improvements across key outcomes, including quality of life, upper limb function, pain, fatigue, and shoulder range of motion. These improvements were primarily observed as within-group changes from baseline to post-intervention, with several studies also reporting superior outcomes compared to conventional rehabilitation.

The findings of this systematic review indicate that technology-assisted rehabilitation interventions are associated with improvements in multiple clinical outcomes, including pain, range of motion, strength, and quality of life. However, these improvements were predominantly observed within groups rather than between groups.

Importantly, several included studies demonstrated significant improvements over time in both intervention and control groups, without statistically significant differences between them [6,20,21,22,23,24]. This suggests that the observed benefits may not be exclusively attributable to the technology-based interventions but could also reflect natural recovery, placebo effects, or the impact of standard rehabilitation.

These findings highlight the need for cautious interpretation of the current evidence. While technology-assisted interventions appear promising, the lack of consistent between-group differences limits the ability to draw firm conclusions regarding their comparative effectiveness [11,29].

The heterogeneity in study designs, outcome measures, and intervention protocols may have contributed to the variability in results. Future research should focus on better-designed randomized controlled trials with adequate power and standardized outcome measures to better determine the added value of technology-assisted interventions compared to conventional rehabilitation.

With respect to upper limb rehabilitation, virtual reality-based interventions appear to provide meaningful functional benefits, particularly through mechanisms related to motor learning and task-specific training. The included studies demonstrated that VR and mixed reality systems facilitate repetitive, goal-directed upper limb movements in an interactive environment, which may enhance neuromuscular coordination and promote motor relearning. These findings are consistent with established rehabilitation principles, where high repetition, feedback, and task specificity are key determinants of functional recovery.

In contrast to conventional physiotherapy or unsupervised home-based programs, technology-enhanced interventions offer real-time feedback and adaptive progression, allowing for more precise adjustment of exercise intensity. This may lead to improved engagement and adherence, as well as more effective stimulation of both muscular strength and functional performance. However, it should be noted that these benefits were not consistently reflected in statistically significant between-group differences, suggesting that while technology may enhance the rehabilitation experience, its superiority over standard care remains uncertain.

Regarding shoulder range of motion, improvements observed across studies appear to be strongly associated with the increased volume and multidirectional nature of movements facilitated by VR- and AR-based systems. These platforms encourage active participation through functional tasks, potentially resulting in greater joint mobility compared to more static or less engaging exercise approaches.

In relation to secondary lymphedema, the available evidence suggests that multimodal interventions combining exercise with conventional components such as compression therapy and manual lymphatic drainage may be more effective than exercise alone. In particular, studies incorporating VR-based exercise alongside complete decongestive therapy demonstrated improvements in both functional outcomes and symptom management. However, due to the limited number of studies specifically targeting lymphedema, definitive conclusions regarding the most effective physiotherapy approach cannot be drawn.

Overall, while virtual reality-based and technology-assisted interventions appear to support upper limb rehabilitation and functional recovery, their added value compared to conventional physiotherapy remains dependent on factors such as exercise design, intensity, and integration with established rehabilitation protocols.

Importantly, the observed benefits appear to be mediated by specific mechanisms related to both exercise prescription and technological integration, rather than the use of technology alone. Technology-enhanced interventions, particularly those incorporating VR, AR, and MR, provided real-time feedback, task-specific training, and adaptive progression of exercise intensity, which are fundamental components of motor learning and rehabilitation [9,12,16].

For example, reductions in pain may be explained by both central and peripheral mechanisms. VR-based interventions create immersive environments that can induce attentional distraction, reducing pain perception through cognitive modulation. At the same time, structured exercise—characterized by controlled repetitions, progressive loading, and improved joint mobility—may contribute to reductions in musculoskeletal pain. This is supported by evidence suggesting that exercise-induced improvements in circulation, tissue flexibility, and neuromuscular function can alleviate pain and enhance functional outcomes [5,11].

Similarly, improvements in upper limb function and shoulder range of motion are likely related to the high volume of task-specific, repetitive movements facilitated by interactive technologies. VR and MR systems require continuous engagement in multi-planar upper limb activities, promoting neuromuscular re-education and motor relearning [9,15]. In addition, MR-based programs incorporating high-intensity interval training (HIIT) and individualized progression ensure that participants exercise at an appropriate intensity, enhancing both muscular strength and endurance [22].

The positive effects on quality of life may be partially attributed to improvements in physical function, but also to enhanced motivation, engagement, and adherence [14]. The interactive and gamified nature of these interventions, combined with real-time performance feedback, may increase patient participation and reduce dropout rates, thereby amplifying the overall effectiveness of rehabilitation.

These findings are consistent with previous systematic reviews in the field. For example, Keshner et al. demonstrated that VR-based rehabilitation enhances functional recovery through improved engagement and motor learning processes [10]. Similarly, Naro et al. emphasized the role of immersive environments and feedback in promoting neuroplasticity and functional gains [9]. In oncology populations, Eid et al. reported that VR-based exercise interventions can improve functional outcomes and quality of life, although their findings were based on heterogeneous cancer populations [14]. Additional systematic reviews have also highlighted the benefits of virtual reality and telerehabilitation in improving functional outcomes and accessibility of rehabilitation services [11,14].

Overall, the present findings suggest that technology-enhanced exercise interventions may provide additional benefits beyond conventional rehabilitation when they are designed to optimize key exercise parameters, including intensity, progression, and task specificity.

## 5. Limitations

This systematic review has several limitations that should be considered when interpreting the findings. A key limitation of the present review is the relatively small number of included studies (n = 6), which may limit the generalizability and strength of the conclusions. This is partly attributable to the strict inclusion criteria, particularly the restriction to randomized controlled trials (RCTs), which was applied to ensure methodological rigor and comparability across studies. However, this approach may have resulted in the exclusion of relevant evidence from pilot, feasibility, quasi-randomized, or hybrid study designs. Given the rapidly evolving nature of technology-assisted rehabilitation, it is possible that emerging interventions have not yet been evaluated in fully powered RCTs. Therefore, the findings of this review should be interpreted with caution, and future research should consider a broader range of study designs to better capture the evolving evidence base.

Secondly, the literature search was limited to three electronic databases (PubMed, CINAHL Complete, and MEDLINE Complete), which may have resulted in the omission of relevant studies indexed in other databases such as Scopus or Web of Science. Although the selected databases provide strong coverage of biomedical and rehabilitation research, the exclusion of additional databases may have contributed to an incomplete representation of the available evidence.

Thirdly, substantial heterogeneity was observed across studies in terms of intervention characteristics, including the type of technology used (e.g., virtual reality, augmented reality, telerehabilitation), exercise modalities (aerobic, resistance, task-specific training), and training parameters (frequency, duration, and intensity) [6,20,21,22,23,24]. This variability may affect the comparability of results across studies.

Different outcome measures were used to assess similar constructs (e.g., quality of life and functional performance), which may affect the comparability of findings. Participant characteristics also varied, with some studies including patients in the early postoperative phase, while others focused on long-term survivors. Moreover, certain studies applied specific age-related inclusion criteria, whereas others did not, potentially influencing baseline functional capacity and responsiveness to intervention.

There was also considerable variability in intervention design, including differences in duration, frequency, and intensity of exercise programs, as well as in the type and implementation of technological modalities. The virtual reality interventions themselves were not standardized, ranging from immersive to non-immersive systems, which may lead to differences in user engagement, motor demands, and overall effectiveness.

Additionally, most studies provided limited or no detailed information regarding tumor subtype or biological characteristics, limiting the ability to explore how underlying disease heterogeneity may influence rehabilitation outcomes.

Beyond these factors, breast cancer itself represents a biologically heterogeneous disease. Emerging evidence suggests that even within traditionally defined subgroups, such as HER2-negative disease, further distinctions (e.g., HER2-low and ultralow expression) may have important implications for treatment response and recovery trajectories [27]. Such biological variability may also influence rehabilitation outcomes, adherence, fatigue levels, and response to technology-assisted interventions.

Another limitation is that several studies presented methodological concerns, particularly related to risk of bias, including lack of participant and therapist blinding and relatively small sample sizes, as assessed using the Cochrane Risk of Bias 2 tool [18]. These factors may reduce confidence in the estimated effects.

Finally, the majority of studies reported short-term outcomes, with limited follow-up data available to evaluate the long-term sustainability of the observed benefits. Additionally, reliance on self-reported outcome measures in some studies may introduce measurement bias.

Future research should focus on conducting high-quality randomized controlled trials with standardized intervention protocols, larger sample sizes, and longer follow-up periods to strengthen the evidence base in this field.

## 6. Conclusions

Technology-enhanced exercise interventions appear to be a promising approach for improving functional outcomes and quality of life among women with breast cancer. However, the current evidence should be interpreted with caution, as improvements were predominantly observed within groups, while consistent between-group differences were not demonstrated across studies.

These findings suggest that, although beneficial effects are reported, the comparative effectiveness of technology-assisted interventions over standard rehabilitation remains inconclusive. Furthermore, the heterogeneity of interventions and methodological limitations across studies highlight the need for more rigorous research.

Future randomized controlled trials should incorporate standardized intervention protocols, consistent outcome measures, and longer follow-up periods to better determine the added value and long-term effectiveness of technology-assisted rehabilitation in breast cancer care.

## Figures and Tables

**Table 1:** Inclusion criteria based on the PECO framework.

Component	Criteria
Population (P)	Adult women with stage I–III breast cancer
Exposure (E)	Technology-enhanced exercise or physiotherapy interventions (e.g., VR, AR, robotics, tele-rehabilitation, digital platforms)
Comparator (C)	Active control group (e.g., conventional physiotherapy or exercise)
Outcomes (O)	At least one of the following: quality of life, mental health (anxiety, depression), upper limb function, fatigue, pain
Study design	Peer-reviewed randomized controlled trials (RCTs)

Abbreviations: VR = virtual reality; AR = augmented reality; RCT = randomized controlled trial.

**Table 4:** GRADE.

Outcome	No. of Studies (RCTs)	Participants	Intervention	Comparison	Certainty of Evidence (GRADE)	Explanation
Quality of Life	4	258	Technology-enhanced exercise (VR, AR, telerehabilitation)	Conventional rehabilitation/home exercise	Low ⊕⊕◯◯	Downgraded due to risk of bias (lack of participant and therapist blinding) and inconsistency (heterogeneity in interventions [VR, AR, telerehabilitation] and outcome measures [FACT-B, SF-36, EORTC, EQ-5D])
Upper Limb Function	5	320	Technology-enhanced exercise	Conventional rehabilitation	Low ⊕⊕◯◯	Downgraded due to risk of bias and inconsistency (variation in intervention type and functional outcome measures such as DASH, grip strength, and performance tests)
Fatigue	2	121	Technology-enhanced exercise	Usual care/home exercise	Very low ⊕◯◯◯	Downgraded due to imprecision (small sample size, limited number of studies) and inconsistency across interventions
Pain	4	240	Technology-enhanced exercise	Conventional rehabilitation	Low ⊕⊕◯◯	Downgraded due to risk of bias (lack of blinding) and imprecision (moderate sample sizes and variability in results across studies)
Shoulder Range of Motion	4	270	Technology-enhanced exercise	Conventional rehabilitation	Low ⊕⊕◯◯	Downgraded due to inconsistency (heterogeneity in exercise protocols and measurement methods) and risk of bias

GRADE scale: ⊕⊕⊕⊕ High certainty: There is high confidence that the true effect lies close to that of the estimate. ⊕⊕⊕◯ Moderate certainty: There is moderate confidence in the effect estimate; the true effect is likely to be close but may be substantially different. ⊕⊕◯◯ Low certainty: Confidence in the effect estimate is limited; the true effect may be substantially different. ⊕◯◯◯ Very low certainty: There is very little confidence in the effect estimate; the true effect is likely to be substantially different.

## Data Availability

No new data were created or analyzed in this study thus data sharing is not applicable.

## Supplementary Materials

The following supporting information can be downloaded at: https://www.mdpi.com/article/10.3390/medicina62040762/s1, File S1: Supplementary File S1 Research Strategy.
