# Supplementary material for: Effectiveness of Emerging Technologies in Physiotherapy for Women with Breast Cancer: A Systematic Review
Source: Medicina (Kaunas). 2026 Apr 15;62(4):762. doi: 10.3390/medicina62040762 (PMC13117946; doi:10.3390/medicina62040762)
Supplement: Supplementary file 1 [file medicina-62-00762-s001.zip › medicina-4237075-supplementary.pdf]

Supplementary File S1. Detailed Search Strategy

A comprehensive literature search was conducted across three electronic databases: **PubMed**, **CINAHL Complete**, and **MEDLINE Complete**.

The search covered the period from **1 January 2010 to March 2026**.

The search strategy was initially developed for PubMed using a combination of keywords and Medical Subject Headings (MeSH). The same conceptual strategy was applied across all databases, with minor adaptations in syntax and indexing terms where necessary.

Search Limits Applied (All Databases)

- Publication date: **01 January 2010 – March 2026**
- Study design: **Randomized Controlled Trials (RCTs)**
- Population: **Humans only**
- Language: **English**
- Publication type: **Peer-reviewed articles**
- Study type: **Primary research only** (reviews, meta-analyses, protocols, and conference abstracts were excluded)

PubMed Search Strategy
**("breast cancer" OR "breast neoplasm*" OR "mammary carcinoma")**
**AND**
**("rehabilitation" OR "physiotherapy" OR "physical therapy" OR "exercise")**
**AND**
**("virtual reality" OR "augmented reality" OR "mixed reality" OR "extended reality"**
**OR "telerehabilitation" OR "digital rehabilitation" OR "technology-assisted" OR "robotics")**
**AND**
**("quality of life" OR "mental health" OR "depression" OR "anxiety"**
**OR "upper limb function" OR "arm function" OR "fatigue" OR "pain")**
**AND**
**("randomized controlled trial")**

Additional Notes

The same core search concepts were applied across all databases to ensure consistency and comparability. Minor adaptations were made where necessary to account for database-specific indexing systems and search interfaces.

To enhance comprehensiveness, the reference lists of included studies were also manually screened to identify any additional eligible studies.
